# Supplementary material for: High-Throughput Sequencing Reveals Transcriptome Signature of Early Liver Development in Goat Kids
Source: Genes (Basel). 2022 May 6;13(5):833. doi: 10.3390/genes13050833 (PMC9141777; doi:10.3390/genes13050833)
Supplement: Supplementary file 1 [file genes-13-00833-s001.zip › Table S1.pdf]

Table S1 Reads of the 25 Libraries.

Sample	Raw Reads	Clean Reads	Error rate (%)	Q20(%)	Q30(%)	GC content (%)
**D1_1**	124706552	122652950	0.0259	97.73	93.26	50.11
**D1_2**	124711436	121783280	0.0261	97.57	93.06	51.29
**D1_3**	121605834	119676092	0.0259	97.65	93.25	50.11
**D1_4**	125007672	120264076	0.0262	97.55	93	50.57
**D1_5**	123313310	120479764	0.0258	97.73	93.39	51.17
**W2_1**	123078860	120859436	0.0258	97.72	93.4	51.45
**W2_2**	125724416	123705754	0.0258	97.73	93.39	50.39
**W2_3**	120567842	118304356	0.026	97.62	93.13	50.06
**W2_4**	162766876	136724132	0.0259	97.7	93.25	50.87
**W2_5**	124667212	122445796	0.0261	97.6	93.12	50.47
**W4_1**	122695698	120410174	0.0262	97.51	92.98	50.46
**W4_2**	119138166	117388094	0.026	97.63	93.16	50.82
**W4_3**	122019306	120185204	0.0261	97.58	93.09	50.54
**W4_4**	124607136	123101152	0.0256	97.81	93.54	51.05
**W4_5**	125188728	123580984	0.0257	97.76	93.39	50.59
**W8_1**	124239242	121955756	0.0254	97.86	93.7	50.97
**W8_2**	120650752	119109088	0.0256	97.83	93.55	50.84
**W8_3**	117802766	115449396	0.0258	97.76	93.38	50.21

**W8_4**	121776430	119620226	0.0258	97.74	93.34	50.8
**W8_5**	125408616	120674068	0.0259	97.69	93.25	50.94
**W12_1**	120588066	119033274	0.0256	97.81	93.55	51.73
**W12_2**	122109328	119799184	0.0267	97.43	92.52	51.05
**W12_3**	122277196	119656710	0.0257	97.78	93.46	50.12
**W12_4**	117731312	114642794	0.0257	97.77	93.44	51
**W12_5**	124226524	122363858	0.0258	97.75	93.33	50.09
