# Supplementary material for: High-Throughput Sequencing Reveals Transcriptome Signature of Early Liver Development in Goat Kids
Source: Genes (Basel). 2022 May 6;13(5):833. doi: 10.3390/genes13050833 (PMC9141777; doi:10.3390/genes13050833)
Supplement: Supplementary file 1 [file genes-13-00833-s001.zip › Table S2.pdf]

Table S2. Summary of sequencing read alignment to the goat reference genome.

Sample	Total reads	Total mapped	Multiple mapped	Unique mapped
**D1_1**	122652950	119526217(97.45%)	11359329(9.26%)	108166888(88.19%)
**D1_2**	121783280	118365352(97.19%)	11693696(9.6%)	106671656(87.59%)
**D1_3**	119676092	116299281(97.18%)	11002117(9.19%)	105297164(87.99%)
**D1_4**	120264076	117157837(97.42%)	12697214(10.56%)	104460623(86.86%)
**D1_5**	120479764	117342955(97.4%)	12589812(10.45%)	104753143(86.95%)
**W2_1**	120859436	117162548(96.94%)	12277063(10.16%)	104885485(86.78%)
**W2_2**	123705754	120370171(97.3%)	12269044(9.92%)	108101127(87.39%)
**W2_3**	118304356	115202551(97.38%)	12025689(10.17%)	103176862(87.21%)
**W2_4**	136724132	132755116(97.1%)	15714255(11.49%)	117040861(85.6%)
**W2_5**	122445796	119027235(97.21%)	12710898(10.38%)	106316337(86.83%)
**W4_1**	120410174	116902441(97.09%)	11231241(9.33%)	105671200(87.76%)
**W4_2**	117388094	114165989(97.26%)	10431646(8.89%)	103734343(88.37%)
**W4_3**	120185204	116771445(97.16%)	12150598(10.11%)	104620847(87.05%)
**W4_4**	123101152	119636922(97.19%)	12345381(10.03%)	107291541(87.16%)
**W4_5**	123580984	120356723(97.39%)	12473458(10.09%)	107883265(87.3%)
**W8_1**	121955756	118574248(97.23%)	13868028(11.37%)	104706220(85.86%)
**W8_2**	119109088	116160798(97.52%)	13173902(11.06%)	102986896(86.46%)
**W8_3**	115449396	112576636(97.51%)	12046959(10.43%)	100529677(87.08%)
**W8_4**	119620226	116392152(97.3%)	13430024(11.23%)	102962128(86.07%)

**W8_5**	120674068	117566378(97.42%)	12965052(10.74%)	104601326(86.68%)
**W12_1**	119033274	115840121(97.32%)	13042473(10.96%)	102797648(86.36%)
**W12_2**	119799184	116198233(96.99%)	11636839(9.71%)	104561394(87.28%)
**W12_3**	119656710	116673515(97.51%)	10534177(8.8%)	106139338(88.7%)
**W12_4**	114642794	111662013(97.4%)	11230955(9.8%)	100431058(87.6%)
**W12_5**	122363858	119161730(97.38%)	10878213(8.89%)	108283517(88.49%)
