# Supplementary material for: High-Throughput Sequencing Reveals Transcriptome Signature of Early Liver Development in Goat Kids
Source: Genes (Basel). 2022 May 6;13(5):833. doi: 10.3390/genes13050833 (PMC9141777; doi:10.3390/genes13050833)
Supplement: Supplementary file 1 [file genes-13-00833-s001.zip › Table S3.pdf]

Table S3 Statistics of known and new transcripts in each group

Sample Name	Known mRNA Num	Novel mRNA Num	All mRNA Num
**D1_1**	19480	7090	26570
**D1_2**	19620	7091	26711
**D1_3**	19620	7070	26690
**D1_4**	19239	7075	26314
**D1_5**	19107	7041	26148
**W2_1**	19335	7089	26424
**W2_2**	19377	7067	26444
**W2_3**	18800	7060	25860
**W2_4**	19430	7062	26492
**W2_5**	19379	7067	26446
**W4_1**	19278	7104	26382
**W4_2**	19501	7093	26594
**W4_3**	19462	7096	26558
**W4_4**	19496	7101	26597
**W4_5**	19519	7093	26612
**W8_1**	18778	7029	25807
**W8_2**	19228	7091	26319
**W8_3**	19235	7049	26284

**W8_4**	18830	7062	25892
**W8_5**	19342	7078	26420
**W12_1**	19438	7062	26500
**W12_2**	19102	7066	26168
**W12_3**	19322	7084	26406
**W12_4**	18927	7071	25998
**W12_5**	19079	7085	26164
